# Considerations for Return to Exercise Following Mild-to-Moderate COVID-19 in the Recreational Athlete

**DOI:** 10.1007/s11420-020-09777-1

**Published:** 2020-08-10

**Authors:** Jordan D. Metzl, Kathryn McElheny, James N. Robinson, Daphne A. Scott, Karen M. Sutton, Brett G. Toresdahl

**Affiliations:** Sports Medicine Institute, Hospital for Special Surgery, 535 E. 70th St., New York, NY 10021 USA

**Keywords:** COVID-19, activity, exercise, recreational athlete

## Abstract

The COVID-19 pandemic has resulted in significant morbidity and mortality around the world. The spectrum of COVID-19 is broad, from clinical disease requiring intensive medical care to less severe symptoms that are treated with supportive care. The majority of COVID-19 cases fall into the mild-to-moderate category, with symptoms lasting less than 6 weeks. Nevertheless, the morbidity from COVID-19 is significant and can affect multiple body systems, most frequently the cardiac, pulmonary, hematologic, musculoskeletal, and gastrointestinal systems. For patients who wish to return to exercise after mild-to-moderate COVID-19, the wide range of disease expression presents a challenge for clinicians seeking to offer counsel. This literature review on return to activity following mild to moderate COVID-19 in the recreational athlete includes evidence-based considerations and recommendations for clinicians in guiding the safest return to activity.

**Electronic supplementary material:**

The online version of this article (10.1007/s11420-020-09777-1) contains supplementary material, which is available to authorized users.

## Introduction

The COVID-19 pandemic has resulted in significant morbidity and mortality throughout the world. As of June 1, 2020, there were 6,057,843 confirmed cases of COVID-19 globally, with 371,166 deaths—numbers that are expected to rise throughout 2020 and considered under-representative of the scope of the disease [49]. The majority of COVID-19 cases are mild to moderate, resulting in symptom resolution within 6 weeks of symptom onset [45]. The most accurate demographic data on COVID-19 rates currently exists in hospitalized patients. Cummings et al. published one of the largest patient cohort studies in the USA, with data from the most extensive COVID-19 domestic outbreak, in New York City [9]. Between March 2 and April 1, 2020, 1150 adults were admitted to New York-Presbyterian hospitals: the median patient age was 62 years, 67% were men, 62% Hispanic or Latino, 19% Black or African American, 12% white, and 3% Asian. Eighty-two percent of patients had at least one chronic illness (the most prevalent were hypertension at 63% and diabetes at 36%), 22% were critically ill, 46% had obesity, and 79% required mechanical ventilation for a median of 18 days.

Despite a relatively low mortality rate, COVID-19 is associated with a wide range of clinical morbidities across body systems [14], most frequently the cardiac, pulmonary, hematologic, musculoskeletal, and gastrointestinal systems.

With a wide range of disease expression and a broad scope of clinical severity, COVID-19 presents a different challenge for each patient returning to activity. This may include the recreational jogger who notices persistent fatigue and the swimmer who suffers ongoing respiratory symptoms. Different from professional athletes who have existing medical programs to guide return to activity, recreational athletes often return to activity in conjunction with a primary care provider. As the number of patients who develop mild-to-moderate COVID-19 continues to grow, and as athletes seek safe ways of re-engaging in a world with continued social distancing [13], clinicians will be increasingly asked to provide return-to-activity guidance. Our goal with this article is to provide an introduction to the considerations for return to activity in recreational athletes who have had mild-to-moderate COVID-19.

### Activity Profiles During the COVID-19 Pandemic

Physical inactivity is a well-recognized risk to physical and mental health that is associated with increased all-cause mortality [5]. The effects of inactivity can rapidly change the body’s metabolism. A 2-week reduction in daily steps from 10,000 to 1500 steps has been shown to impair insulin sensitivity and lipid metabolism and increase visceral fat in otherwise healthy adults [30].

During the early stages of the COVID-19 pandemic, there was a documented decrease in physical activity including daily walking volume and step count [12]. The effects of this relative inactivity are important to consider when examining the risk for chronic disease expression due to relative inactivity.

### Effects of COVID-19 by Body System

The system-based approach to COVID-19 is a helpful way to evaluate the effects of this disease. We have chosen to address the most frequently reported body-system effects including cardiac, pulmonary, musculoskeletal, hematologic, and gastrointestinal systems. The effects of COVID-19 to other areas, including the neurological, psychological, dermatological, and renal systems, have also been described [44].

### Cardiac System

Various cardiac complications have been associated with COVID-19, including arrhythmia, myocarditis, and acute myocardial injury [1, 17, 40, 46]. Systemic inflammation, direct injury of cardiomyocytes, “cytokine storm,” interstitial fibrosis, and hypoxia are some of the proposed mechanisms of the multifactorial pathophysiology [25]. Arrhythmias in this setting can also be attributed to treatment with azithromycin, hydroxychloroquine, and some antivirals that can cause QT prolongation [35]. The prevalence of cardiac involvement among nonhospitalized patients is unknown. Symptomatology of cardiac involvement in COVID-19 is inconsistent and may present as chest pain, palpitations, or fatigue. Diagnostic testing for acutely ill patients with known or suspected COVID-19 includes resting 12-lead electrocardiography (ECG), testing of cardiac troponin and natriuretic peptide levels, and echocardiography, when clinically warranted [27].

While regular exercise improves cardiovascular health in the long-term, each session of exercise stresses the heart and can trigger potentially lethal arrhythmias in the context of underlying cardiovascular disease [2]. Many nonhospitalized individuals with COVID-19 will likely not develop cardiac manifestations and be able to safely return to exercise. Despite this, care must be taken to ensure the absence of persistent cardiac complications related to COVID-19 prior to returning to exercise.

Return-to-sport recommendations have been published for competitive athletes with COVID-19 [25, 37]. These vary in the extent of recommended testing prior to returning to play. Hull et al. suggest return to play for athletes with COVID-19 after resting from sport for at least 10 days after symptom onset and 7 days after symptom resolution without additional testing [23]. Baggish et al. in a blog post recommend that prior to returning to training after COVID-19, all competitive athletes undergo a history and physical examination, including those who were asymptomatic; for those who were symptomatic but not severely enough to be hospitalized, ECG should be performed as well [2]. Phalen et al. recommend a 2-week period of rest for competitive athletes and highly active individuals who test positive for COVID-19 but are asymptomatic, followed by a gradual return to exercise under the guidance of a medical team; for athletes who are symptomatic but not hospitalized, they recommend 2 weeks of rest after resolution of symptoms followed by evaluation with troponin testing, ECG, and echocardiography [37].

As for recreational athletes, Emery et al. recommend resumption of moderate intensity activity without additional testing only in those patients without pre-existing cardiac conditions who had mild-to-moderate symptoms and have completely recovered [11]. Barker-Davies et al. are more conservative and recommend 2 to 3 weeks of rest (less than 3 metabolic equivalents) after symptom resolution, but in a patient with myocarditis associated with COVID-19, they recommend rest from exercise for 3 to 6 months followed by testing with biomarkers, echocardiography, rhythm monitoring, and exercise testing [3].

### Pulmonary System

Early in the course of the pandemic, researchers noted that fever and cough were the dominant presenting symptoms of COVID-19. Pneumonia appeared to be the most frequent serious manifestation of infection, characterized primarily by fever, cough, dyspnea, and bilateral infiltrates on chest imaging [21].

While the majority of people with COVID-19 have no symptoms or very mild respiratory symptoms and recover within 5 to 7 days, there has been an apparent heightened risk of deterioration in respiratory status between days 7 and 14. During this time, worsening symptoms and manifestations of COVID-19 can develop, requiring higher levels of care, including intensive care unit (ICU) admission and intubation [21]. Acute respiratory distress syndrome (ARDS) is the major pulmonary complication in patients with severe disease. In a study of 138 patients in Wuhan, China, ARDS developed in 20% of patients at a median of 8 days after symptom onset and mechanical ventilation was implemented in 12.3% [45]. In studies from the USA, up to 23% of hospitalized patients have required mechanical ventilation [36].

According to the World Health Organization (WHO), the median time from symptom onset to clinical recovery is approximately 2 weeks, and 3 to 6 weeks for patients with severe or critical disease [47]. Many young people recover without sequalae; however, establishing guidelines on how and when to return to activity for those with more severe disease or are older and slower to recover has become an important action item.

Evidence-based recommendations for the resumption of sports and exercise after COVID-19 are currently limited and continue to evolve [10]. Guidance published in the *Lancet Respiratory Medicine* recommend prolonged rest after infection, 10 or more days from symptom onset plus 7 days from symptom resolution [23]. These recommendations, however, do not address those with more severe disease manifestations.

For those who survive ARDS, there is no data specific to recovery from COVID-19, but in 2003, Herridge et al. evaluated 109 survivors of ARDS at 3, 6, and 12 months after ICU discharge [20]. Lung volume and spirometry measurements were normal by 6 months, but carbon monoxide diffusion capacity remained low throughout the 12-month follow-up period. No patients required supplemental oxygen at 12 months, but 6% of patients had arterial oxygen saturation levels below 88% during exercise. The distance walked in 6 min, the primary outcome measure of the study, improved over 12 months but remained lower than the predicted value [20]. Until we have more definitive and specific data relating to ARDS secondary to COVID-19, it is recommended that those with severe pulmonary manifestations be followed clinically by a pulmonary specialist, with return to exercise based on recovery.

Overall, a careful monitoring of respiratory symptoms and a gradual return to activity are warranted in the recreational athlete who has suffered respiratory symptoms of COVID-19. If patients have an underlying history of pulmonary disease, the attention to pulmonary symptoms should be emphasized.

### Musculoskeletal System

There are currently few known orthopedic issues directly related to COVID-19. Bones, articular cartilage, synovium, and skeletal and smooth muscle all express one or some combination of angiotensin-converting enzyme 2 (ACE2) and type 2 transmembrane serine protease (TMPRSS2) receptors serving as potential targets of SARS-CoV-2, the virus that causes COVID-19. There is also potential for damage secondary to increased inflammatory cytokines [10]. The most common musculoskeletal complaints from COVID-19 are myalgia and arthralgia. Myalgia was a presenting symptom in 15% of patients with COVID-19 [16]. The myalgias are usually self-limited and resolve over a period of a few days to 2 weeks. As with any viral myositis, treatment of COVID-19-related myalgia is supportive and includes heat, ice, topical analgesia, and stretching. Intense exercise should be avoided in those experiencing myalgia or muscle fatigue. Acetaminophen may also be helpful for pain control. Fatigue is commonly reported in addition to myalgias and may last for weeks. While sarcopenia, loss of bone mineral density, and osteonecrosis were seen with SARS, it is unclear if these were the result of the viral infection itself or of treatment with corticosteroids. It remains to be seen if similar clinical patterns will be seen with COVID-19.

The use of nonsteroidal anti-inflammatory drugs (NSAIDs) in patients with COVID-19 is controversial, with initial concerns that NSAID use increased risk of more severe infection in COVID-19 patients [16]. However, there have been no studies to date that conclusively show a correlation of COVID-19 illness severity with NSAID use. Furthermore, as NSAIDs are known to decrease prostaglandins, pro-inflammatory cytokines, and interleukin 6, there may be a role for NSAIDs in the treatment of the cytokine storm, although this has not yet been clinically demonstrated [38]. The WHO does not currently recommend against NSAID use in patients with COVID-19 [48]. Corticosteroid injections should be used with caution during the COVID-19 pandemic [39, 50], and when necessary we suggest using betamethasone or dexamethasone [18, 28].

From a musculoskeletal perspective, return to exercise in a patient recovering from COVID-19 should be guided by symptoms while symptomatic patients should avoid intense exercise. Once symptoms have resolved, a gradual progression to normal activity can begin. While there is currently a paucity of data related to returning to exercise after COVID-19, low-intensity exercise for 1 week is suggested before returning to more rigorous exercise [3]. Due to inactivity and deconditioning, those recovering from infection may be at increased risk of injury as they return to exercise [6].

### Hematologic System

Although the risk factors have yet to be fully understood, some patients with COVID-19 are at increased risk for hypercoagulability and subsequent thrombosis. The most frequent thrombotic complications in hospitalized patients include deep venous thrombosis and pulmonary embolism [15, 19, 34]. Large vessel strokes, myocardial injury, and microvascular thrombosis of the toes have also been demonstrated; the true incidence of these prothrombotic complications among various presentations and severities of COVID-19 have not been clearly defined [33, 43]. A French prospective multi-center trial with 150 critically ill patients revealed a pulmonary embolism incidence of 16.7% despite prophylactic anticoagulation [19]. Similarly, a Dutch study including 184 ICU patients reported a venous thromboembolism (VTE) rate of 27% despite prophylaxis [26]. Interestingly, US centers have shown much lower rates of VTE; of 13 (3.3%) of 393 COVID-19 hospitalized patients in New York City who developed VTE, ten were mechanically ventilated, but three did not require ventilation in an ICU [14].

The pathogenesis of COVID-19-associated hypercoagulability remains unknown [41]. It is hypothesized that hypoxia and systemic inflammation may lead to high levels of inflammatory cytokines and subsequent activation of the coagulation pathway [4]. Laboratory abnormalities commonly include mild thrombocytopenia; elevated D-dimer levels; increased fibrinogen, fibrin, and fibrin degradation products; and a prolonged prothrombin time. D-dimer levels that are significantly elevated (greater than six times the upper limit of normal) have been associated with increased risk of death [32].

Unfortunately, less is known about clotting tendencies in the asymptomatic or mildly symptomatic person with COVID-19 who does not require hospitalization. In non-hospitalized patients, there is currently no data to support obtaining routine coagulation values. Similarly, there is insufficient evidence to recommend for or against routine DVT screening with imaging in COVID-19 patients without signs or symptoms of a clot, regardless of coagulation markers (if obtained). Each of the studies described relied upon clinical suggestive findings of thrombosis to prompt additional work-up.

While there is evidence of increased likelihood of multiorgan failure in patients with sepsis who develop coagulopathy, there is insufficient data to demonstrate that any particular anticoagulation treatment will improve outcomes in patients with COVID-19 [36]. The American Society of Hematology recommends prophylactic dose low molecular weight heparin in all hospitalized COVID-19 patients who are without active bleeding with a platelet count greater than 25 × 10^9^/L and fibrinogen greater than 0.5 g/L [29]. Therapeutic anticoagulation is not required unless atrial fibrillation or VTE is documented. The efficacy of full therapeutic anticoagulation for critically ill COVID-19 patients without documented VTE is currently under study. Routine post-discharge VTE prophylaxis is not recommended in COVID-19 patients with the exception of consideration in certain high-risk individuals based on age, D-dimer level, and modified International Medical Prevention Registry on Venous Thromboembolism (IMPROVE)-VTE score [4]. It is recommended that patients currently receiving anticoagulation or antiplatelet therapy continue their medications as prescribed if they are diagnosed with COVID-19 and that anticoagulants or antiplatelet therapy not be intitiated in the non-hospitalized COVID-19 population [8].

Hypercoagulability is of concern in the athlete returning to recreational sports after COVID-19. Lower extremity deep venous thrombosis (DVT) often clinically presents as calf pain with or without erythema and edema. While there are several acute and chronic musculotendinous etiologies for calf pain in an athlete, during the COVID-19 pandemic VTE should be considered, as should the potential protection offered by mobility. Virchow’s triad describes three broad categories of factors that are thought to contribute to thrombosis: hypercoagulability, stasis, and endothelial injury/dysfunction. Immobility and travel are known risk factors for VTE formation given their role in stasis and hypercoagulability [24]. While exercise is not currently recommended in someone acutely symptomatic with COVID-19 for several pulmonary and cardiac reasons [19], low-intensity exercise, or at least avoidance of prolonged sitting, might offer some protection from hypercoagulability tendencies in the non-hospitalized asymptomatic or mildly symptomatic COVID-19 patient. However, there is insufficient data to currently support this hypothesis.

### Gastrointestinal System

Some patients with COVID-19 develop gastrointestinal symptoms such as vomiting, nausea, anorexia, and diarrhea. The percentage of patients experiencing these symptoms is variable. Stanford researchers evaluated 116 patients with COVID-19 and found that 31.9% experienced gastrointestinal symptoms, with 22% having nausea and vomiting and 12% having diarrhea [7]. A high percentage of these patients (22%) also experienced loss of appetite. The main considerations for athletes who have had gastrointestinal manifestations as part of COVID-19 include hydration and energy availability upon return to training once medically cleared. Fluid and calorie intake should be monitored throughout the symptomatic phase of the illness as well as during symptom resolution upon return to activity in the recreational athlete.

### Return to Activity Considerations

After severe acute respiratory syndrome (SARS), caused by SARS-CoV-1, became a global pandemic in 2003 [31, 42], patients were followed after return to exercise. The history of the return to exercise in these patients offers insight into return to activity considerations in recreational athletes who have recovered from COVID-19. In the SARS patients, 110 survivors with confirmed SARS-CoV-1 infection were evaluated and studied by Hui et al. in Hong Kong at both 3- and 6-month intervals [22]. The exercise capacity and health status of the survivors were considerably lower than that of a normal population at 6 months. Thirty percent had abnormal chest radiographs, 15.5% of patients had significant impairment in surface area for gas exchange. The functional disability appeared disproportionate to the impairment lung function and may have been related to muscle deconditioning and steroid myopathy [22].

As we learned in the 2003 SARS pandemic, COVID-19 patients should be followed closely, especially in the first 3 to 6 months as they return to exercise programs (Table 1). This is true for athletes who have had COVID-19 to any degree. In the initial phases of return to exercise after recovery from mild infection, we recommend a gradual guided activity modification plan such as the 50/30/20/10 rule developed by the National Strength and Conditioning Association and Collegiate Strength and Conditioning Coaches Association Joint committee for use over a 4-week period [6]. The conditioning volume for the first week is reduced by at least 50% of the normal exercise load, followed by 30%, 20%, and 10% in the following 3 weeks if comfortable at the end of each week. This would be adjusted by the severity of the disease and may require a graduated return to activity occurring over many months rather than weeks [6].

**Table 1:** Considerations and recommendations for recreational athletes returning to activity after COVID-19

**Considerations**	
Each patient with COVID-19 is unique. Although general patterns in COVID-19 have been reported, there is a wide variance of disease expression.	
Each patient with COVID-19 recovers at a unique rate. There is currently no algorithm guiding a patient’s stepwise return to activity.	
The severity of disease appears to affect the duration of recovery, although this has yet to be proven.	
Return to activity after COVID-19 should be guided by a body-system approach that includes the cardiac, pulmonary, hematologic, musculoskeletal, and gastrointestinal systems.	
Clinicians should advise patients to return to activity in a slow, gradual, stepwise manner.	
Patients should be given instructions to speak with their health care provider should they develop symptoms in the body systems listed above.	
**Recommendations**	
Exercise should not resume if a patient with COVID-19 has persistent fever, dyspnea at rest, cough, chest pain, or palpitations.	
Any COVID-19 patient with an underlying cardiovascular or pulmonary condition should consult a physician prior to resumption of exercise, even if asymptomatic.	
An otherwise healthy patient with a self-limited course of COVID-19 who has been asymptomatic for 7 days may begin resuming physical activity at 50% of normal intensity and volume.	
Consultation with a physician is recommended if patients who have had COVID-19 develop chest pain, fever, palpitations, or dyspnea on the resumption of exercise.	

Given the rapidly evolving scientific landscape surrounding COVID-19, guidance for safe return to recreational exercise is likely to change. Consequently, there is need for providing a framework and preliminary recommendations for safe return to exercise based on current knowledge.

## Electronic Supplementary Material

ESM 1 (PDF 1224 kb)

ESM 2 (PDF 1224 kb)

ESM 3 (PDF 1224 kb)

ESM 4 (PDF 1224 kb)

ESM 5 (PDF 1224 kb)

ESM 6 (PDF 1224 kb)
